# FFP: joint Fast Fourier transform and fractal dimension in amino acid property-aware phylogenetic analysis

**DOI:** 10.1186/s12859-022-04889-3

**Published:** 2022-08-19

**Authors:** Wei Li, Lina Yang, Yu Qiu, Yujian Yuan, Xichun Li, Zuqiang Meng

**Affiliations:** 1 School of Computer, Electronics and Information, Guangxi University, Nanning, China; 2 Guangxi Normal University for Nationalities, Chongzuo, China

**Keywords:** Phylogenetic analysis, Amino acid property, Fast Fourier transform, Dissociation constant, Higuchi’s fractal dimension, Protein sequence similarity, Multi-sequence alignment

## Abstract

**Background:**

Amino acid property-aware phylogenetic analysis (APPA) refers to the phylogenetic analysis method based on amino acid property encoding, which is used for understanding and inferring evolutionary relationships between species from the molecular perspective. Fast Fourier transform (FFT) and Higuchi’s fractal dimension (HFD) have excellent performance in describing sequences’ structural and complexity information for APPA. However, with the exponential growth of protein sequence data, it is very important to develop a reliable APPA method for protein sequence analysis.

**Results:**

Consequently, we propose a new method named FFP, it joints FFT and HFD. Firstly, FFP is used to encode protein sequences on the basis of the important physicochemical properties of amino acids, the dissociation constant, which determines acidity and basicity of protein molecules. Secondly, FFT and HFD are used to generate the feature vectors of encoded sequences, whereafter, the distance matrix is calculated from the cosine function, which describes the degree of similarity between species. The smaller the distance between them, the more similar they are. Finally, the phylogenetic tree is constructed. When FFP is tested for phylogenetic analysis on four groups of protein sequences, the results are obviously better than other comparisons, with the highest accuracy up to more than 97%.

**Conclusion:**

FFP has higher accuracy in APPA and multi-sequence alignment. It also can measure the protein sequence similarity effectively. And it is hoped to play a role in APPA’s related research.

## Background

Proteins perform vital roles in countless biological processes, they help to build the structure of living organisms. Generally, proteins’ three-dimensional structure depends on primary amino acid sequence and determines their biological function [1]. Sequence analysis based on biomolecular data can reduce the time and cost of traditional laboratory experiments for protein family identification, function prediction and gene annotation [2]. Due to the explosive growth of genome sequence data, it is necessary to find a reliable algorithm for sequence analysis [3].

Detecting similar fragments between sequences is the core idea of multi-sequence alignment (MSA) [4, 5], whose reliability directly affects protein phylogenetic analysis in revealing the distance relationship among different species [6]. Existing MSA algorithms can be divided into two categories: alignment-based and alignment-free algorithms. Compared with the former algorithms [7, 8], alignment-free has lower computing complexity and better visualization. Among these alignment-free algorithms, the graphic representation of protein is one of the most effective and commonly used ways. Hamori and Ruskin first applied it to biomolecular sequences data [9]. After that, many different graphical representation methods of protein sequences have been proposed for further sequence analysis. El-Lakkani [10] represent protein sequences using 3D adjacency matrix, which is an improvement based on 2D adjacency matrix representation [11]. Gupta et al. [12], Wu [13], Yang [14] represent protein sequences and carried out similarity analysis based on hydrophobicity values of the amino acid.

In addition, the physical and chemical properties of amino acids play a significant role in the functional and structural formation of proteins. Thus, there are some methods based on properties have been proposed. The literature [15–20] reduced 20 amino acids to 4–12, and they divided the amino acids into 4–12 groups based on amino acids hydrophobicity and isoelectric points. This simplification may result in the loss of biological information. Yu [21] used the hydrophobicity, dissociation constant and accessible surface area of amino acids to combine with spherical coordinates to represent protein sequences. Mu [22] transformed sequences into 578 numerical vectors for protein phylogenetic analysis. Rout et al. [23] proposed EightyDVec for protein phylogenetic analysis based on the physicochemical properties of amino acids.

Moreover, some signal processing algorithms (Discrete Fourier Transform, Fast Fourier Transform(FFT), Higuchi’s fractal dimension (HFD)) have also been introduced into protein sequence analysis. Hou et al. [24] proposed a sequence similarity analysis method based on Discrete Fourier Transform and Dynamic Time Warping that has a high time calculation cost and it can only compare time domain sequence, not in the frequency domain [25]. Compared with Discrete Fourier Transform, FFT can save exponential computing time. FFT is good at capturing the frequency content of the signal, which may contain the essence of the data. Guo proposed a method to classify G-protein coupled receptors based on FFT [26]. Chen proposed a random projection method based on FFT for self-interacting proteins prediction [27]. Fractal dimension describes the complexity of geometric objects. Smits used HFD to monitor the complexity of brain activity [28]. There exists similarity between the whole and part of the protein sequence, so they can be represented by fractal curve. Hu [29] calculated the similarity between protein sequences based on box-counting dimension.

Although FFT and HFD have been widely used, no one used them together for Amino acid property-aware phylogenetic analysis (APPA), which refers to the phylogenetic analysis based on amino acid property encoding, and it is an effective method to study the similarity and functional relationships between protein sequences [30]. The primary sequence is represented by 20 amino acid letters, and this representation cannot be processed directly and needs to be converted to numbers [31]. Effective amino acid digital coding is related to the overall performance of the model, which is usually called feature extraction or amino acid coding scheme [32]. The property of amino acids plays a decisive role in the formation of protein structure and function. Therefore, amino acid property encoding is used in this paper, and we aim to discuss the application of FFT and HFD in APPA.

**Fig. 1:**
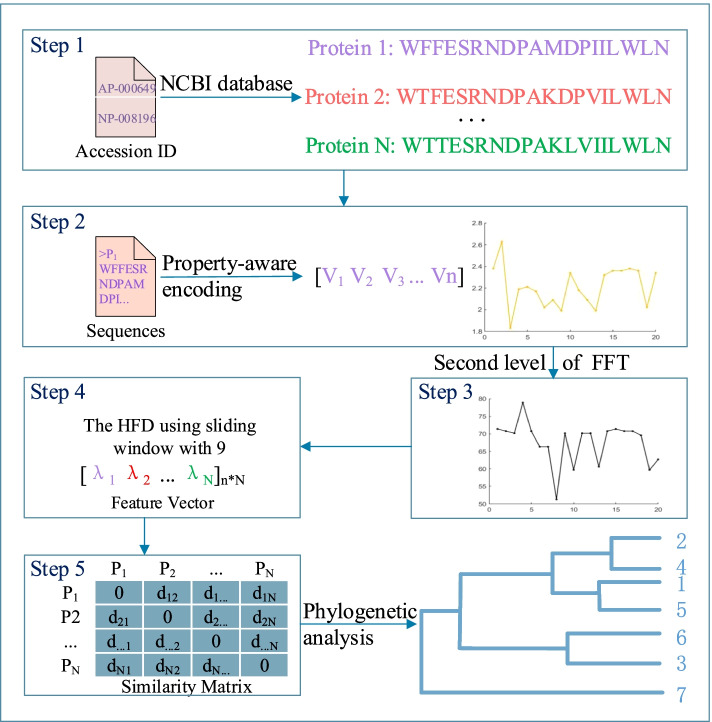
Overall steps of sequence comparison algorithm. *Step* 1 The primary amino acid sequences (Protein 1,...,Protein N) are queried from the NCBI database according to the Accession ID of proteins. *Step* 2 Each amino acid letter in P$_j$ is mapped to its attribute value and obtain the curvilinear representation of P$_j$. *Step* 3 Calculate the Discrete Fourier transform of P$_j$ using the FFT. *Step* 4 Calculate the feature vectors of Step 3 based on HFD. *Step* 5 Calculate the distance between pairwise protein sequences using Cosine function. Finally, phylogenetic trees can be constructed based on single linkage

In this paper, we present FFP, it is a hybrid method for APPA. Above all, the primary amino acid sequence is converted into digital sequence using the pK$_a$(COOH) value, which is critical for the dissociation constant. In previous works, the hydrophobicity of amino acids is the most used, as an equally important dissociation constant, it is rarely used. Next, the feature vector of each protein is generated by integrating FFT and HFD. Then the distance matrix is obtained by the cosine function, the shorter the distance between two species, the more similar they are. (Details are shown in Fig. 1 and *Materials and Methods*). Finally, FFP is applied to the phylogenetic analysis of a set of ND6 proteins and three sets of $\beta$-globin proteins with different sizes, respectively. And the results are also compared with previous works to demonstrate the effectiveness of our method.

## Results

To demonstrate the accuracy of our method, we used FFP for phylogenetic analysis on four groups of frequently-used protein sequences. The protein data information used in this section is given in Table 1. We use trial and error to set the FFT level to 2, the sliding window width to 9 by observing the phylogenetic tree, which is obtained by the linkage and dendrogram function in Matlab. For comparison, we also chose the same data set with some existing distance-based phylogenetic algorithms, they are based on Neighbor-Joining algorithm ( [33]), UPGMA algorithm [34] ( [19, 20] and [35]), Euclidean distance algorithm ( [18]) and Jeffrey’s and Matusita distance algorithm ( [29]). All of these methods are alignment-free. In order to illustrate the performance of our method more effectively, we also compare with ClustalW, the representative of alignment-based methods. The phylogenetic tree built by ClustalW is implemented using UPGMA algorithm in the MEGA [36]. All protein data used in the experiment are obtained from NCBI database [37].

### Phylogenetic analysis of 8 ND6 protein sequences

This dataset contains 8 ND6 protein sequences from different species, the sequence details are given in Table 1 : *ND6Set*. A 159 $\times$ 8 feature vector was obtained by FFT and HFD.

**Table 1:** A summary of the four data sets used in the experiment

Species/Set	Accession ID	Length	Species/Set	Accession ID	Length
1. *ND6Set*			2. *10-BetaSet*		
Human	YP_003024037	174	Human	AAA16334	147
Gorilla	NP_008223	174	Gorilla	P02024	147
C.Chimp	NP_008197	174	Gibbon	P02025	146
Wallaroo	NP_007405	167	G.Panda	P18983	147
Harbor Seal(H.Seal)	NP_006939	175	Goose	P02117	146
Gray Seal(G.Seal)	NP_007080	175	Swan	P68945	146
Rat	AP_004903	172	Goat	AAA30913	145
Mouse	NP_904339	172	Sheep	NP_001091117	145
			Bovine	CAA25111	145
			Bison	P09422	145
3. *11-BetaSet*			4. *17-BetaSet*		
Human	AAA16334	147	Human	ALU64020	147
Lemur	AAA36822	147	Gorilla	P02024	147
Mouse	ADD52696	147	Chimpanzee (Chimp)	P68873	147
Goat	AAA30913	145	Cattle	CAA25111	145
Rabbit	CAA24251	147	Banteng	BAJ05126	145
Chimpanzee	P68873	147	Goat	AAA30913	145
Gorilla	P02024	147	Sheep	ABC86525	145
Rat	CAA33250	147	European hare (E.Hare)	CAA68429	147
Bovine	CAA25111	145	Rabbit	CAA24251	147
Opossum	AAA30976	147	House mouse (H.Mouse)	ADD52660	147
Gallus	CAA23700	147	Western wild mouse	ACY03394	147
			Spiny mouse (S.Mouse)	ACY03377	147
			Norway Rat (N.Rat)	CAA29887	147
			Opossum	AAA30976	147
			Guttata	CH46399	147
			Gallus	CAA23700	147
			Muscovy duck(M.Duck)	CAA33756	147

**Fig. 2:**
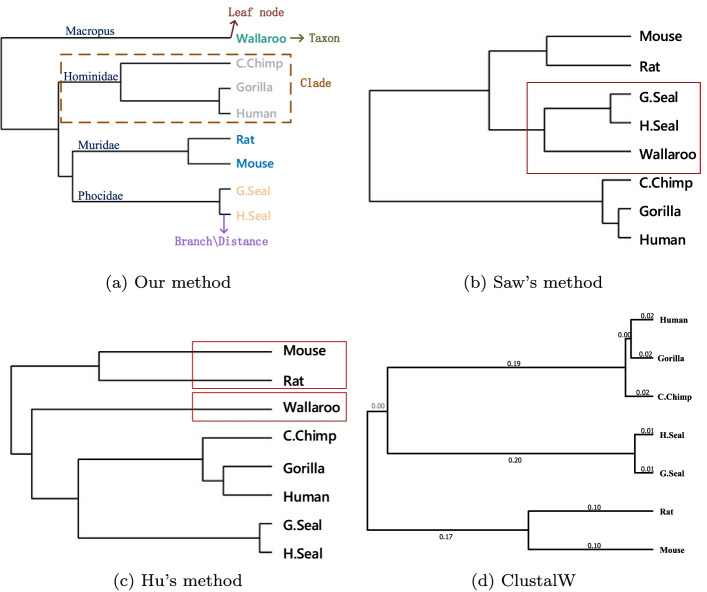
Phylogenetic trees of *ND6Set* constructed by **a** Our method using FFP, **b** Saw’s method, **c** Hu’s method and **d** ClustalW

The cosine function was used to calculate the distance matrix of eight ND6 protein sequences of mammals, the matrix is filled in Table 2. The smaller the value in the matrix, the smaller the distance between species and the more similar they are. And the phylogenetic tree was constructed by single linkage, as shown in the Fig. 2. The horizontal axis (branch) is the similarity between species, and the vertical axis is eight different species. The shorter the branches, the smaller distance the two sequences and the closer the two species.

As shown in Fig. 2a, these proteins were correctly divided into four groups, and each category was highlighted in a different color: they are **Hominidae** (Human, Gorilla and C.Chimp), **Phocidae** (Harbor seal and Gray seal), **Muridae** (Rat and Mouse) and **Macropus** (Wallaroo). In terms of molecular evolution, Human and Gorilla shared the common ancestor millions of years ago. The closer the species are to each other, the shorter their evolutionary distance. From the biochemical point of view, there are minimal different sites in the primary amino acid sequence between them, so they are clustered firstly. The same is true for other species. Moreover, chimpanzees are more closely related to humans than are gorillas [38], Wallaroo is the most distant from the other seven mammals. These results are consistent with known evolutionary facts.

**Table 2:** The distance matrix of *ND6Set* by FFP

	Human	Gorilla	C.Chimp	Wallaroo	H.Seal	G.Seal	Rat	Mouse
Human	0	0.0002	0.0017	0.0122	0.0061	0.0065	0.0078	0.0085
Gorilla	0.0002	0	0.0019	0.0128	0.0070	0.0071	0.0080	0.0091
C.Chimp	0.0017	0.0019	0	0.0098	0.0032	0.0037	0.0054	0.0065
Wallaroo	0.0122	0.0128	0.0098	0	0.0070	0.0066	0.0064	0.0062
H.Seal	0.0061	0.0070	0.0032	0.0070	0	0.0006	0.0051	0.0048
G.Seal	0.0065	0.0071	0.0037	0.0066	0.0006	0	0.0046	0.0041
Rat	0.0078	0.0080	0.0054	0.0064	0.0051	0.0046	0	0.0016
Mouse	0.0085	0.0091	0.0065	0.0062	0.0048	0.0041	0.0016	0

Phylogenetic trees constructed by previous studies [29, 33], and ClustalW are shown in Fig. 2b–d, respectively. Figure 2b also correctly classifies eight species into four groups, but incorrectly connects Wallaroo to the Seal branch. Wallaroo is the farthest from the other seven species. In Fig. 2c , the phylogenetic tree given by Hu shows Muridae (Rat and Mouse) are the most distant of the eight species, they are closer to Hominidae (Human, Gorilla and C.Chimp) than Wallaroo. Figure 2d is the phylogenetic tree constructed by ClustalW [7] using Mega [36] package, which constructs the phylogenetic tree by UPGMA (Unweighted Pair Group Method with Arithmetic Mean) method, it is one of the most recognized methods in protein MSA [39], the difference between it and Fig. 2a is which family is closer to Phocidae (H.Seal and G.Seal), Hominidae or Muridae (Rat and Mouse). According to the Encyclopedia Britannica [40], Rat and Mouse are insectivores, G.Heal and H.Seal are carnivores, Human, Gorilla and C.Chimp are omnivorous, thus, Muridae is closer to Phocidae than Hominidae. And Wallaroo is herbivorous, so it is the most distant from the other seven mammals. He’s [18] result showed that Muridae branch is closer to Hominidae than Phocidae.

We also calculated the Correlation coefficient (CC) between existing works (including ours, Ref. [29, 33]) with ClustalW’s result. The CC of Human is calculated by the first row of our distance matrix in Table 2 and the first row of the matrix obtained by ClustalW and so on. In statistical analysis, if CC *c* between variable *A* and variable *B* satisfies $c_{0.05}(n-2)<|c| \le c_{0.01}(n-2)$ (*n* is the number of variables), this is to say that *A* and *B* in linear correlation. In this part, n=8, so when $0.707<|c| \le 0.834$, it’s in linear correlation, and when $|c| > 0.834$, it’s in strongly linear correlation. The calculated CC results are filled in Table 3. It can be seen that our results are all strongly linear correlation with ClustlW except Wallaroo, but it’s still in linear correlation, and our result’s correlation coefficients with ClustalW’s are all higher than Ref. [33]. However, some of [29]’s CCs with ClustalW’s are higher than ours, his clustering of Wallaroo was inaccurate.

**Table 3:** The correlation coefficients for *ND6Set* between our, Saw’s [33] and Hu’s [29] method with ClustalW

	Human	Gorilla	C.Chimp	Wallaroo	H.Seal	G.Seal	Rat	Mouse
Our CC	0.9754	0.9715	0.8881	0.7431	0.9016	0.8866	0.9107	0.8544
[33]’s CC	0.8469	0.8832	0.8554	0.8653	0.7602	0.7847	0.8112	0.7536
[29]’s CC	0.9707	0.9700	0.9648	0.9806	0.9436	0.9481	0.8890	0.9197

### Phylogenetic analysis of 10 $\beta$-globin protein sequences

**Fig. 3:**
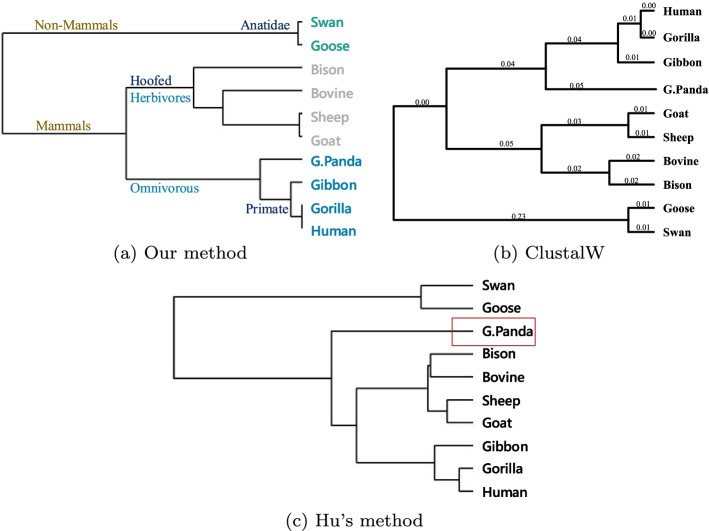
Phylogenetic trees of *10-BetaSet* constructed by **a** Our method, **b** ClustalW and **c** Hu’s method

This dataset used 10 $\beta$-globin from different species (see Table 1: *10-BetaSet* for details). The distance matrix using cosine function is shown in Table 4. The smaller the value between them, the more similar the protein sequences are, and the more closely related the species are. To more intuitively describe this relationship, we constructed the phylogenetic tree (Fig. 3a:) of these 10 species using the single linkage.

**Table 4:** The distance matrix of *10-BetaSet* by FFP

	Human	Gorilla	Gibbon	G.Panda	Goose	Swan	Goat	Sheep	Bovine	Bison
Human	0	0.0000	0.0001	0.0004	0.0032	0.0032	0.0028	0.0028	0.0018	0.0034
Gorilla	0.0000	0	0.0001	0.0004	0.0032	0.0032	0.0028	0.0028	0.0018	0.0034
Gibbon	0.0001	0.0001	0	0.0003	0.0031	0.0031	0.0028	0.0027	0.0019	0.0035
G.Panda	0.0004	0.0004	0.0003	0	0.0035	0.0036	0.0024	0.0023	0.0016	0.0032
Goose	0.0032	0.0032	0.0031	0.0035	0	0.0000	0.0038	0.0037	0.0029	0.0041
Swan	0.0032	0.0032	0.0031	0.0036	0.0000	0	0.0038	0.0037	0.0030	0.0041
Goat	0.0028	0.0028	0.0028	0.0024	0.0038	0.0038	0	0.0000	0.0011	0.0008
Sheep	0.0028	0.0028	0.0027	0.0023	0.0037	0.0037	0.0000	0	0.0011	0.0008
Bovine	0.0018	0.0018	0.0019	0.0016	0.0029	0.0030	0.0011	0.0011	0	0.0011
Bison	0.0034	0.0034	0.0035	0.0032	0.0041	0.0041	0.0008	0.0008	0.0011	0

As shown in Fig. 3a, these species are divided into two main groups: mammals and non-mammals. Among the mammals, they are classified into **Primate**: Human (Hominiade) and Gorilla (Hominiade) and Gibbon (Hylobatidae), **Carnivora**: Giant panda and **Hoofed**: Sheep, Goat, Bison and Bovine. Non-mammals include **Anatidae**: Swan and Goose.

In terms of molecular evolution, Swan and Goose are non-mammals, so they are the most evolutionarily distant from the other mammals. And they have the minimal different sites in their amino acid sequences, so their distance is near to 0. Among them, Human and Gorilla are the most similar, they are belong to Hominiade. Gibbon is similar in size to apes (Gorillas, Chimpanzees, etc.) and with no tail, just longer arms and thicker hair. In addition, Human, Gorilla and Gibbon are belong to the primate group of Mammals. In terms of eating habits, Human, Gorilla and Gibbon are omnivorous. In accordance evolution aspect, G.Panda’s ancestors were carnivores millions of years ago and gradually became omnivorous over the course of biological evolution, although its main diet is bamboo. Furthermore, Sheep, Goat, Bison and Bovine are herbivores. Given that, G.panda is closer to Human than Hoofed. These conclusions are almost consistent with ClustalW (Fig. 3b). The only difference is that our phylogenetic tree didn’t cluster Bison and Bovine together preferentially. In Fig. 3c, the phylogenetic tree constructed by Ref. [29], G.Panda is the farthest species from the other seven mammals, which could be due to the loss of biological information.

The CC of our method with ClustalW’s and Hu’s [29] with ClustalW’s can be found in Table 5. In this part, n=10, so when $0.632<|c| \le 0.735$, it’s in linear correlation, and when $|c| > 0.765$, it’s in strongly linear correlation. It can be seen that our results are all strongly linear correlation with ClustlW. Half of the results are higher than Hu’s, the CC of G.Panda of Hu’s is only about 0.6, which is considered to be low correlated.

**Table 5:** The CC of our method with ClustalW’s and Hu’s [29] with ClustalW’s for *10-BetaSet*

	Human	Gorilla	Gibbon	G.Panda	Goose	Swan	Goat	Sheep	Bovine	Bison
Our CC	0.8942	0.8828	0.8788	0.9070	0.9675	0.9701	0.9397	0.9402	0.9383	0.8436
[29]’s CC	0.8721	0.8908	0.8504	0.6484	0.9439	0.9458	0.9597	0.9610	0.9502	0.9586

### Phylogenetic analysis of 11 $\beta$-globin protein sequences

**Fig. 4:**
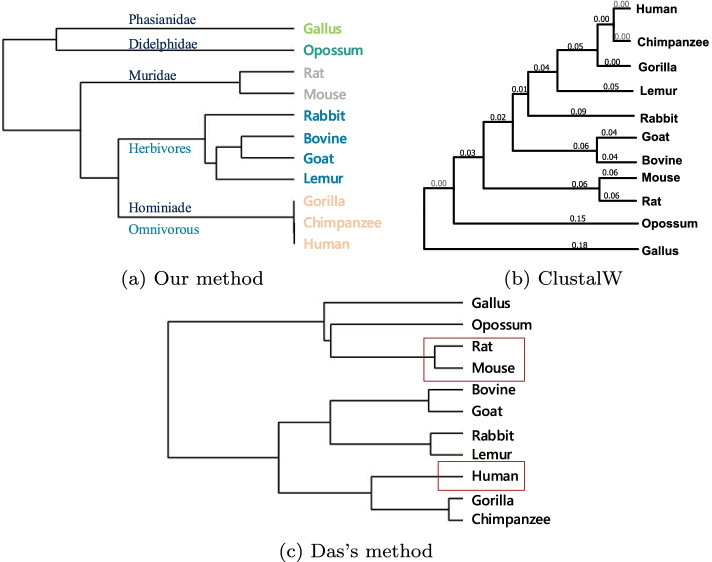
Phylogenetic trees of *11-BetaSet* constructed by **a** Our method, **b** ClustalW and **c** Das’s method

In this experiment, we choose $\beta$-globin protein sequences from 11 different species, and their detailed information is shown in Table 1: *11-BetaSet*. The distance matrix obtained by cosine function is filled in Table 6. It can be seen in Table 6, the distance between Human and C.Chimp is near to 0, which means they are the most similar of these species. The next smallest distance is Gorilla and Human and so on. According to these, the constructed phylogenetic tree is shown in Fig. 4a.

**Table 6:** The distance matrix of *11-BetaSet* by FFP.

	Human	Lemur	Mouse	Goat	Rabbit	C.Chimp	Gorilla	Rat	Bovine	Opossum	Gallus
Human	0	0.0052	0.0075	0.0054	0.0046	0	0.0000	0.0084	0.0050	0.0077	0.0107
Lemur	0.0052	0	0.0058	0.0032	0.0039	0.0052	0.0052	0.0054	0.0028	0.0076	0.0081
Mouse	0.0075	0.0058	0	0.0044	0.0070	0.0075	0.0075	0.0038	0.0073	0.0121	0.0107
Goat	0.0054	0.0032	0.0044	0	0.0031	0.0054	0.0054	0.0062	0.0020	0.0084	0.0076
Rabbit	0.0046	0.0039	0.0070	0.0031	0	0.0046	0.0046	0.0076	0.0027	0.0106	0.0085
C.Chimp	0	0.0052	0.0075	0.0054	0.0046	0	0.0000	0.0084	0.0050	0.0077	0.0107
Gorilla	0.0000	0.0052	0.0075	0.0054	0.0046	0.0000	0	0.0084	0.0050	0.0077	0.0107
Rat	0.0084	0.0054	0.0038	0.0062	0.0076	0.0084	0.0084	0	0.0065	0.0108	0.0113
Bovine	0.0050	0.0028	0.0073	0.0020	0.0027	0.0050	0.0050	0.0065	0	0.0074	0.0079
Opossum	0.0077	0.0076	0.0121	0.0084	0.0106	0.0077	0.0077	0.0108	0.0074	0	0.0093
Gallus	0.0107	0.0081	0.0107	0.0076	0.0085	0.0107	0.0107	0.0113	0.0079	0.0093	0

Figure 4a shows that Human, Chimpanzee and Gorilla are the closest among 11 species because they all belong Hominiade. Next are Goat and Bovine (Hoofed), Lemur (Lemuridae) and Rabbit (Leporidae), they are clustered together since they are herbivorous. The next branch is Muridae: Rat and Mouse. Last is Opossum (Didelphidae) and Gallus (Phasianidae). It seems that Opossum and Gallus should not be grouped together because Gallus is non-mammal. Figure 4b is the phylogenetic tree of ClustalW, which clustered Rabbit and Lemur to the human branch. In Fig. 4c, the result in Ref. [35], didn’t cluster Human and Chimpanzee firstly, which didn’t fit the biochemical and molecular evolution facts and it indicated that the Muridae (Rat and Mouse) is closer to Opossum and Gallus.

### Phylogenetic analysis of 17 $\beta$-globin protein sequences

The data set for the final set of experiments was $\beta$-globin sequences from 17 different species. The accession ID is filled in Table 1: *17-BetaSet*. After calculating of FFP, a 137 $\times$ 17 feature vector was obtained. The choice of distance function is cosine, the distance matrix is shown in Table 7.

**Table 7:** The distance matrix of *17-BetaSet* by FFP.

	Human	Gorilla	Chimp	Cattle	Banteng	Goat	Sheep	E.Hare	Rabbit	H.Mouse	W.Mouse	S.Mouse	N.Rat	Opossum	Guttata	Gallus	M.Duck
Human	0	0.0000	0	0.0050	0.0051	0.0054	0.0049	0.0057	0.0046	0.0075	0.0083	0.0092	0.0084	0.0077	0.0102	0.0107	0.0121
Gorilla		0	0.0000	0.0050	0.0051	0.0054	0.0049	0.0057	0.0046	0.0075	0.0083	0.0092	0.0084	0.0077	0.0102	0.0107	0.0121
Chimp			0	0.0050	0.0051	0.0054	0.0049	0.0057	0.0046	0.0075	0.0083	0.0092	0.0084	0.0077	0.0102	0.0107	0.0121
Cattle				0	0.0002	0.0020	0.0005	0.0044	0.0027	0.0073	0.0077	0.0083	0.0065	0.0074	0.0072	0.0079	0.0082
Banteng					0	0.0023	0.0005	0.0045	0.0028	0.0074	0.0079	0.0085	0.0061	0.0069	0.0071	0.0080	0.0076
Goat						0	0.0015	0.0042	0.0031	0.0044	0.0048	0.0053	0.0062	0.0084	0.0076	0.0076	0.0089
Sheep							0	0.0042	0.0023	0.0073	0.0076	0.0081	0.0063	0.0079	0.0070	0.0075	0.0079
E.Hare								0	0.0023	0.0072	0.0078	0.0078	0.0080	0.0088	0.0080	0.0078	0.0092
Rabbit									0	0.0070	0.0075	0.0085	0.0076	0.0106	0.0086	0.0085	0.0102
H.Mouse										0	0.0010	0.0019	0.0038	0.0121	0.0111	0.0107	0.0128
W.Mouse											0	0.0014	0.0054	0.0133	0.0115	0.0111	0.0134
S.Mouse												0	0.0052	0.0121	0.0086	0.0082	0.0102
N.Rat													0	0.0108	0.0115	0.0113	0.0126
Opossum														0	0.0081	0.0093	0.0083
Guttata															0	0.0006	0.0009
Gallus																0	0.0016
M.Duck																	0

**Fig. 5:**
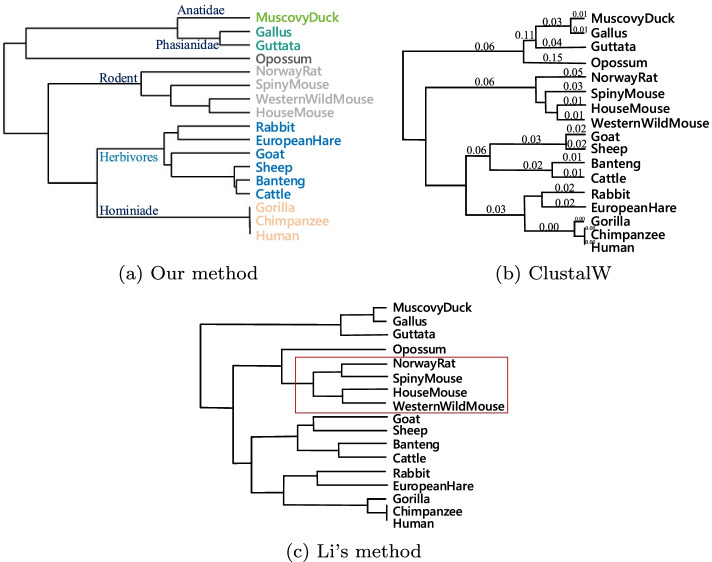
Phylogenetic trees of *17-BetaSet* constructed by **a** Our method, **b** ClustalW and **c** Li’s method

It is clear from Table 7 that the distance between Human, Chimp and Gorilla is the shortest. After four decimal places, the distance between Human and Chimp is 0, which means they are the most similar. The same and more precise information can be obtained from the phylogenetic tree constructed using the single method in Fig. 5a.

In Fig. 5a, it clusters Human, Gorilla and Chimpanzee firstly. The second branch is Banteng, Cattle, Sheep and Goat, they are Hoofed. Next is family Leporida, Rabbit and European hare. And Rodent: House mouse, Western wild mouse, Spiny mouse and Norway Rat. Finally is family Phasianidae: Guttata and Gallus and family Anatidae: MuscovyDuck. It shows that our results are basically consistent with ClustalW (Fig. 5b) and Ref. [20] (Figure 5c). Nevertheless, Fig. 5c thought that Opossum are closer to Rodent than Human and other species. Opossum is the most distant species from the other thirteen mammals.

### Extended experiments

**Fig. 6:**
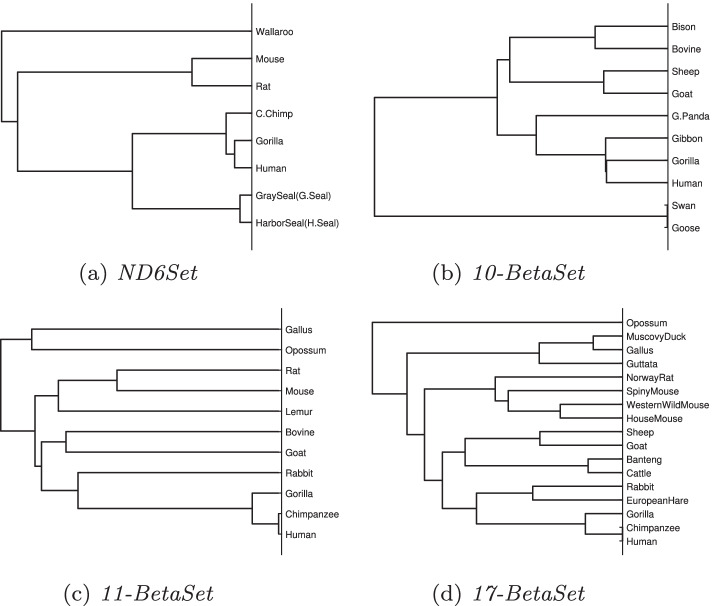
Phylogenetic trees of four test sets using hydrophobicity encoding based on FFP

**Fig. 7:**
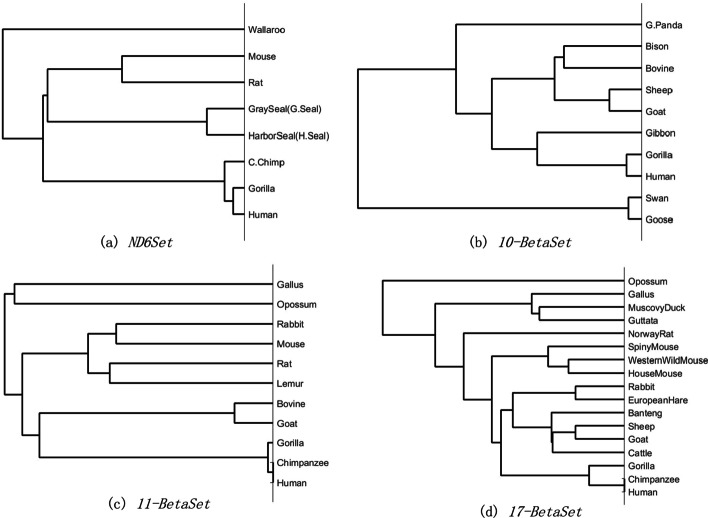
Phylogenetic trees of four test sets using basicity encoding based on FFP

**Fig. 8:**
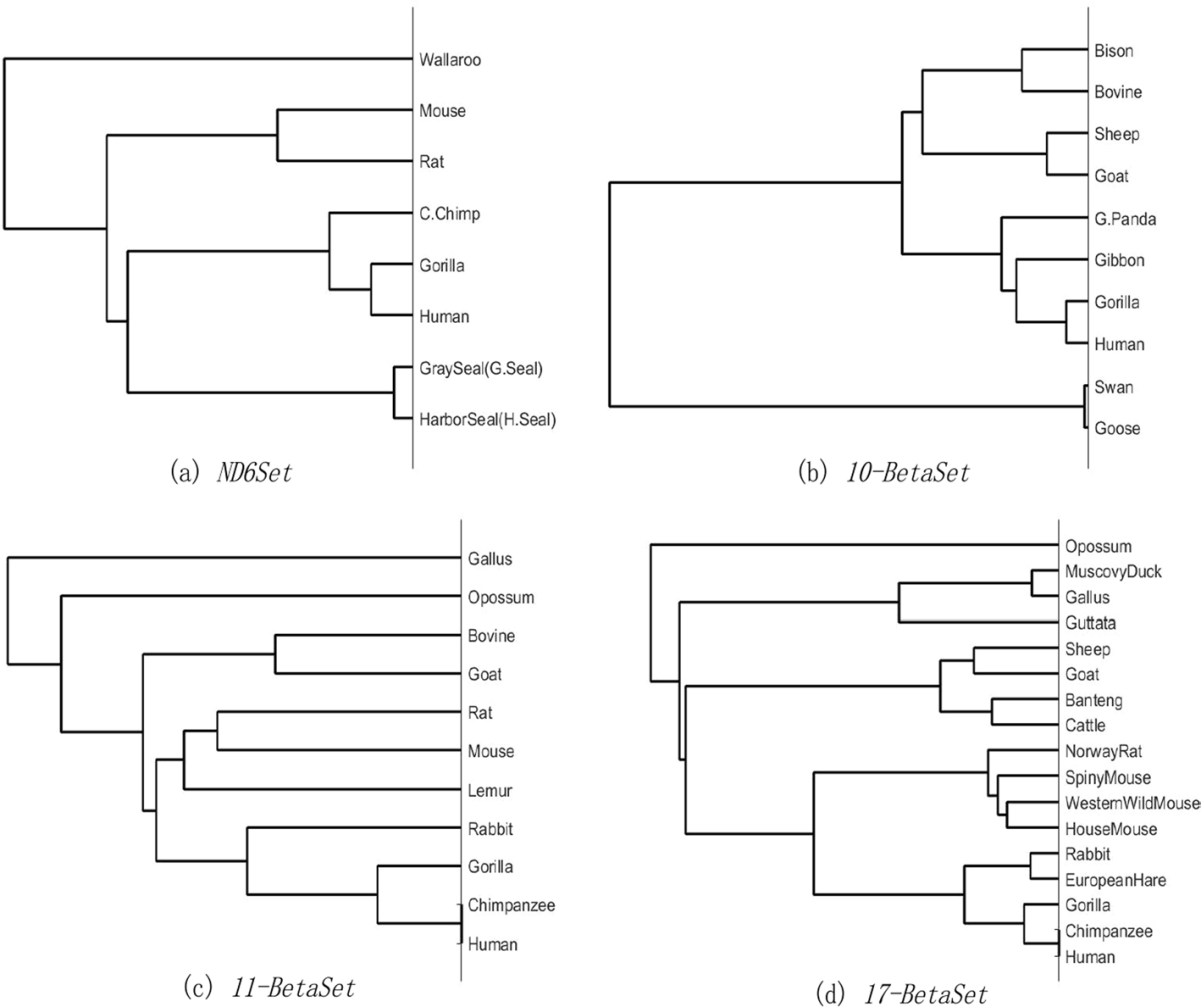
Phylogenetic trees of four test sets using relative molecular mass encoding based on FFP

In this part, the hydrophobic value, basicity coefficient and relative molecular weight of amino acids were used to encode the primary amino acid sequences in four data sets, respectively. After applying FFP to each data set, the constructed phylogenetic trees are shown in Figs 6, 7 and 8, which are also highly similar to our previous tree in **Results**. Hence, it can be concluded that FFP we proposed in this paper is robust.

## Discussion

In this paper, a hybrid method called FFP for APPA was proposed. The differences between FFP and existing works are as follows: (1) In the step of drawing protein sequence curve, we choose dissociation constant among the rich physical and chemical properties of amino acids to encode the protein sequence, which determines the acidity and basicity, making the constructed protein sequence curves more reliable. (2) When extracting the numerical features of protein curves, we use FFT to decompose the initial N-point sequence into a series of short sequences to obtain the potential information in the sequence. (3) To extract more accurate features, we use HFD as the next step of the FFT, which can get information about the geometrical structure.

We tested FFP on one group of ND6 sequences and three groups of globin sequences with different sizes in the experimental part. The results show that FFP is effective for APPA. This method can play a powerful role in the protein classification and the prediction of functional structure. In the meanwhile, FFP still has some improvements to make. For instance, the current FFP algorithm describes protein sequences only based on the properties of amino acids, which may not be comprehensive. Our next research topic will be how to effectively utilize the structural information of proteins and combine it with their properties. In addition, our subsequent work will improve FFP so that it can be more accurate when analyzing protein families with a more significant number.

## Conclusions

Based on the dissociation constant of amino acids, we proposed a hybrid algorithm named FFP for APPA. We tested one group of ND6 sequences and three groups of globin sequences with different sizes in the experimental part. The results show that FFP is effective for proteins phylogenetic analysis. This method can play a powerful role in protein sequences similarity analysis and functional structure prediction. In addition, our subsequent work will improve the algorithm so that it can be more accurate when analyzing protein families with a more significant number.

## Methods

### Data selection and feature extraction

The four different data sets used in the experiment are as follows:

(i) *ND6Set*: NADH Dehydrogenase 6 (ND6) protein sequences of 8 species.
(ii) *10-BetaSet*: $\beta$-globin protein sequences of 10 species.
(iii) *11-BetaSet*: $\beta$-globin protein sequences of 11 species.
(iv) *17-BetaSet*: $\beta$-globin protein sequences of 17 species.

All sequence information are obtained from the NCBI (National Center for Biotechnology Information) database [37], including amino acid sequence, definition, accession ID, sequence length and source.

As the primary structure of protein, amino acid sequence has an important influence on the structure and function of protein. In general, each amino acid is represented by a corresponding letter: A, C, D, E, F, G, H, I, K, L, M, N, P, Q, R, S, T, V, W and Y. The rich properties of amino acids play a decisive role in the structure formation and function of proteins [41]. Isoelectric point (pI) is one of the most important and commonly used properties of amino acids, and the dissociation constant of –COOH (pK$_a$(COOH)) is closely related to pI, it reflects the ionized state of –COOH in solutions. So pK$_a$(COOH) values are used as features to represent amino acids and vectorial protein sequence is obtained. Detailed mappings of each amino acid and their pK$_a$(COOH) values are listed in Table 8.

**Table 8:** Information and feature values of 20 amino acids

	code	pK$_a$(COOH)		code	pK$_a$(COOH)
Ala	A	2.34	Met	M	2.28
Cys	C	1.71	Asp	N	2.02
Asp	D	2.09	Pro	P	1.99
Glu	E	2.19	Glu	Q	2.17
Phe	F	1.83	Arg	R	2.17
Gly	G	2.34	Ser	S	2.21
His	H	1.82	Thr	T	2.63
Ile	I	2.36	Val	V	2.32
Lys	K	2.18	Trp	W	2.38
Leu	L	2.36	Tyr	Y	2.2

Take two short sequences of Saccharomyces cerevisiae as an example, and their sequences are

Protein I (P1): WTFESRNDPAKDPVILWLNGGPGCSSLTGL

Protein II (P2): WFFESRNDPAMDPIILWLNGGPGCSSFTGL

**Fig. 9:**
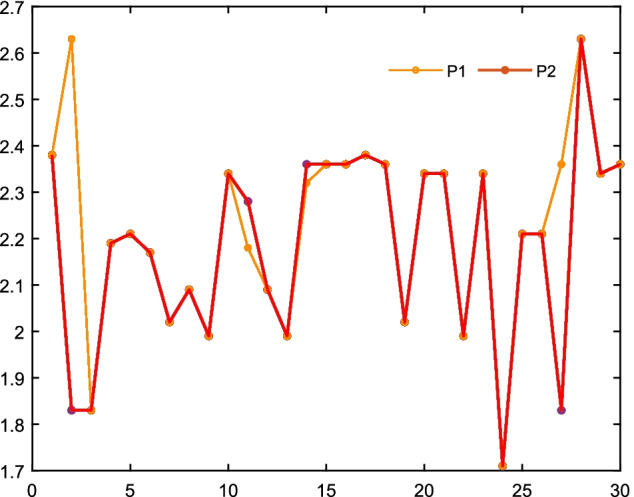
The feature curves of P1 and P2. The x-coordinate means the i-th amino acid, and the y-coordinate is the pK$_a$(COOH) value corresponding to the i-th amino acid. The four positions of the yellow circle are where the two sequences differ

Their feature curves are shown in Fig. 9. The four positions of the yellow circle are where the two sequences differ.

### Fast Fourier transform

As a widely used tool in signal analysis, Discrete Fourier Transform (DFT) and its extension has also been applied to biological sequence analysis [42–47]. Using DFT can discover hidden signal information without loss in the time domain. Fast Fourier Transform (FFT) is a fast algorithm for DFT. The time complexity of DFT is $\Theta \left( n^{2}\right)$, however, the time complexity of FFT is only $\Theta \left( nlgn\right)$. After feature extraction in the previous section, protein sequence S = {$s_1, s_2 \ldots s_N$} can be represented by P = {$p_1, p_2 \ldots p_N$}, and N is the length of protein S, $s_i$ is the i-th amino acid of S and $p_i$ is pK$_a$(COOH) value corresponding to $s_i, i = 1...N$.

The DFT of sequence P = {$p_1, p_2 \ldots p_N$} at frequency *k* is

1 $$\begin{aligned} F(k)=DFT\left[ p_{(n)}\right] =\sum _{n=0}^{N-1} p_{(n)} e^{-jnk\frac{2\pi }{N}}, \quad k=0,1, \ldots , N-1; j=\sqrt{-1}. \end{aligned}$$

**Fig. 10:**
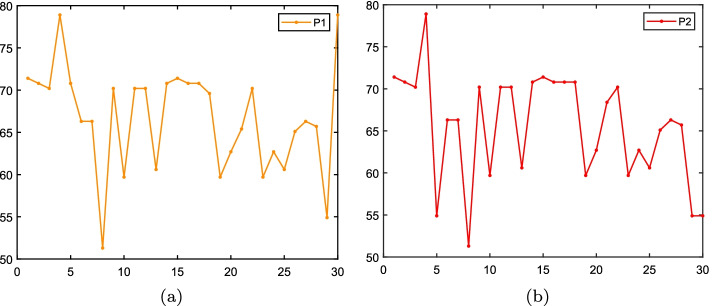
The FFT of P1 and P2. The x-coordinate means the i-th amino acid, and the y-coordinate is FFT using second level

Figure 10 shows the FFT of P1 and P2.

### Higuchi’s fractal dimension

The concept of fractal [48] is very important for the study of non-linear objects. Fractal dimension is an important approach to study fractal, which includes information about the complexity of fractal objects [49]. Hausdorff dimension is one of the oldest and most important fractal dimensions, it gave a new form to the usual concepts of length and area, and it formed the basic theoretical model of other fractal dimensions. However, in practical application, Hausdorff dimension is difficult to calculate or estimate by general calculation method [14]. In contrast, Box counting dimension [50] is more practical and convenient because it is the only dimension that can be computed with a limited range of scales [49]. In order to apply Box counting dimension to digital image processing more conveniently, scholars also put forward Minkowski dimension [50].

However, in some signal and image processing applications, the calculation of Box counting dimension is time-consuming. Thus, some approximate algorithms for fractal dimension were proposed. Higuchi’s fractal dimension (HFD) [51] can provide a better measure of signal complexity when there are few data points available [52]. Therefore, HFD has been widely used in biomedical signal and image processing [53–55]. HFD can be calculated as follows. Suppose that $Z=\left\{ z_{1}, z_{2}, \ldots , z_{M}\right\}$ is a M sample data sequence, and its sub-sequence can be represented as [56]:

2 $$\begin{aligned} Z_{n}^{m}:\left\{ z(n), z(n+m), z(n+2m), \ldots , z\left( n+\left\lfloor \frac{M-n}{m}\right\rfloor m\right) \right\} , n=1...m, \end{aligned}$$

and symbol $\lfloor *\rfloor$ is floor operation, *n* is initial position, *m* means the number of sub-sequences. Now, set *M* =6 and *m* =2, then two sub-sequences are obtained:

$$\begin{aligned} \begin{array}{l} Z_{1}^{2}:\{z(1), z(3), z(5)\}, Z_{2}^{2}:\{z(2), z(4), z(6)\}. \end{array} \end{aligned}$$

The length of each sub-sequence is:

3 $$\begin{aligned} \begin{aligned} H_{n}^m = \sum _{i=1}^{\left\lfloor \frac{M-n}{m} \right\rfloor }\left| z(n+im)-z(n+(i-1)m)\right| (M-1)\div \left\lfloor \frac{M-n}{m} \right\rfloor m^2 \end{aligned} \end{aligned}$$

In addition, we also choose sliding window combine with HFD, a feature vector of length $M-d+1$ can be obtained. $H_{n}^m$ can be rewritten to:

4 $$\begin{aligned} \begin{aligned} H_{n}^j(m) = \sum _{i=1}^{\left\lfloor \frac{d-n}{m} \right\rfloor }\left| z(n+im+j-1)-z(n+(i-1)m+ {j-1)}\right| (d-1)\div \left\lfloor \frac{d-n}{m} \right\rfloor m^2 \end{aligned} \end{aligned}$$

where *d* means the window width, $j = 1...M-d+1$ and $n=1...m$. Then the average length is:

5 $$\begin{aligned} {H}^j(m)=\frac{1}{m}\sum _{n=1}^{m}H_{n}^j(m). \end{aligned}$$

**Fig. 11:**
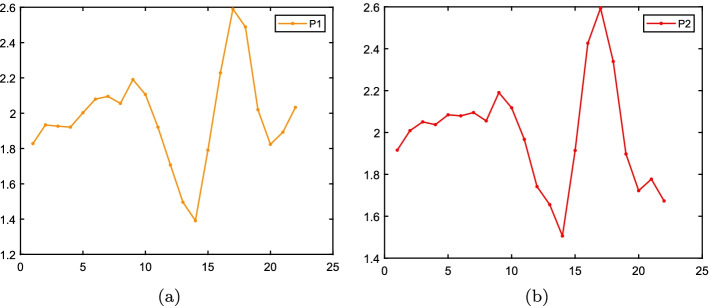
The HFD of P1 and P2 in Fig. 10 using window width 9. The x-coordinate means the j-th window, and the y-coordinate is HFD

Finally, the HFD of window *j* is:

6 $$\begin{aligned} f^{j*}=argmin\sum _{m=1}^{M}(f log(\frac{1}{m})-log({H}^j(m))+b)^{2}. \end{aligned}$$

where *b* is the bias, and the final vector could be represented as $F^{*}=\left\{ f^{1*}, f^{2*}, \ldots , f^{(M-d+1)*}\right\}$. Fig. 11 is the HFD of Fig. 10 with window width 9.

### Similarity function

Phylogenetic tree construction depends heavily on the selection of similarity function. After experimental comparison, cosine similarity is selected in this paper. It evaluates the similarity of two vectors by calculating the cosine of the angle between them [14]. Its calculation formula is as follows:

7 $$\begin{aligned} C=\cos (\theta )=\frac{A \cdot B}{\Vert A\Vert \Vert B\Vert }=\frac{\sum _{i=1}^{n} A_{i} \times B_{i}}{\sqrt{\sum _{i=1}^{n}\left( A_{i}\right) ^{2}} \times \sqrt{\sum _{i=1}^{n}\left( B_{i}\right) ^{2}}}, -1\le C \le 1 \end{aligned}$$

where $A_{i}$ and $B_{i}$ represent the components of vectors *A* and *B*. Finally, the method for linkage is single, it clusters samples according to the distance from near to far.
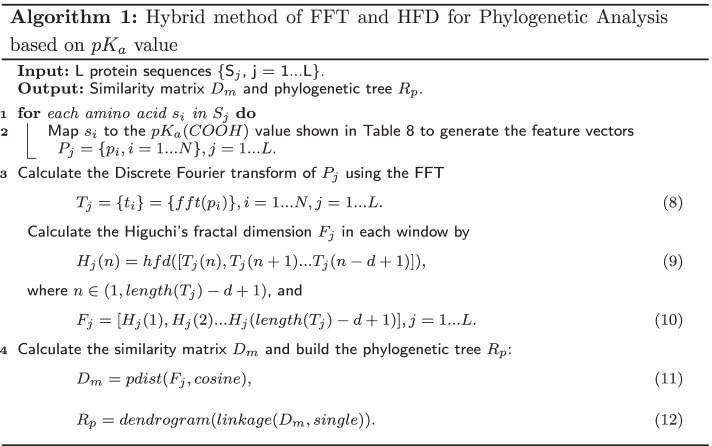


### Algorithm summary

The specific algorithm of FFP is shown in Algorithm 1, it is the concrete implementation of the overall step diagram (Fig. 1).

## Data Availability

All data generated or analysed during this study are included in this published article and its supplementary information files.

## Abbreviations

FFP: Joint fast Fourier transform and fractal dimension in amino acid property-aware phylogenetic analysis
APPA: Amino acid property-aware phylogenetic analysis
FFT: Fast Fourier transform
HFD: Higuchi’s fractal dimension
MSA: Multi-sequence alignment
UPGMA: Unweighted pair group method with arithmetic mean
CC: Correlation coefficient
ND6: NADH dehydrogenase 6
DFT: Discrete Fourier transform
